# *c*-3,*t*-3-Dimethyl-4-oxo-*r*-2,*c*-6-diphenyl­piperidine-1-carboxamide

**DOI:** 10.1107/S1600536809041579

**Published:** 2009-10-23

**Authors:** M. Thenmozhi, T. Kavitha, S. Ponnuswamy, M. Jamesh, M. N. Ponnuswamy

**Affiliations:** a Centre of Advanced Study in Crystallography and Biophysics, University of Madras, Guindy Campus, Chennai 600 025, India; b Department of Chemistry, Government Arts College (Autonomous), Coimbatore 641 018, India

## Abstract

In the title compound, C_26_H_26_N_2_O_2_, the piperidinone ring adopts a distorted boat conformation. The two phenyl rings substituted at positions 2 and 6 of the piperidinone ring occupy axial and equatorial orientations, which are approximately perpendicular to each other [89.14 (8)°]. The phenyl­carbamoyl group adopts an extended conformation. The crystal structure is stabilized by inter­molecular C—H⋯O inter­actions.

## Related literature

For general background to the pharmaceutical activity of piperidine derivatives, see: Mobio *et al.* (1989 ▶); Palani *et al.* (2002 ▶). For hybridization, see: Beddoes *et al.* (1986 ▶). For hydrogen-bond motifs, see: Bernstein *et al.* (1995 ▶). For ring conformational analysis, see: Cremer & Pople (1975 ▶); Nardelli (1983 ▶).
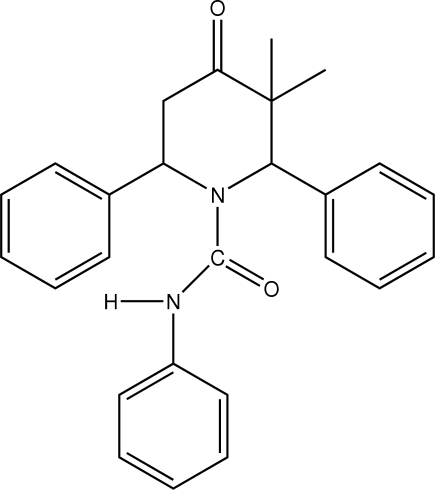


## Experimental

### 

#### Crystal data

C_26_H_26_N_2_O_2_
*M* *_r_* = 398.49
Triclinic, 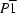

*a* = 9.6648 (2) Å
*b* = 10.7938 (3) Å
*c* = 11.4233 (3) Å
α = 101.303 (2)°
β = 90.158 (1)°
γ = 113.191 (1)°
*V* = 1069.91 (5) Å^3^
*Z* = 2
Mo *K*α radiation
μ = 0.08 mm^−1^
*T* = 293 K
0.12 × 0.12 × 0.10 mm

#### Data collection

Bruker Kappa APEXII area-detector diffractometer
Absorption correction: multi-scan (*SADABS*; Sheldrick, 2001 ▶) *T*_min_ = 0.991, *T*_max_ = 0.992
26776 measured reflections
6362 independent reflections
4483 reflections with *I* > 2σ(*I*)
*R*_int_ = 0.027

#### Refinement

*R*[*F* ^2^ > 2σ(*F* ^2^)] = 0.045
*wR*(*F* ^2^) = 0.129
*S* = 1.03
6362 reflections
278 parameters
1 restraint
H atoms treated by a mixture of independent and constrained refinement
Δρ_max_ = 0.23 e Å^−3^
Δρ_min_ = −0.16 e Å^−3^

### 

Data collection: *APEX2* (Bruker, 2004 ▶); cell refinement: *SAINT* (Bruker, 2004 ▶); data reduction: *SAINT*; program(s) used to solve structure: *SHELXS97* (Sheldrick, 2008 ▶); program(s) used to refine structure: *SHELXL97* (Sheldrick, 2008 ▶); molecular graphics: *ORTEP-3* (Farrugia, 1997 ▶); software used to prepare material for publication: *SHELXL97* and *PLATON* (Spek, 2009 ▶).

## Supplementary Material

Crystal structure: contains datablocks global, I. DOI: 10.1107/S1600536809041579/bt5059sup1.cif

Structure factors: contains datablocks I. DOI: 10.1107/S1600536809041579/bt5059Isup2.hkl

Additional supplementary materials:  crystallographic information; 3D view; checkCIF report

## Figures and Tables

**Table 1:** Hydrogen-bond geometry (Å, °)

*D*—H⋯*A*	*D*—H	H⋯*A*	*D*⋯*A*	*D*—H⋯*A*
C18—H18⋯O2^i^	0.93	2.56	3.4488 (17)	161
C20—H20*B*⋯O1^ii^	0.96	2.52	3.4520 (17)	162

Symmetry codes: (i) 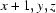; (ii) 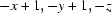.

**Fig. 1.**
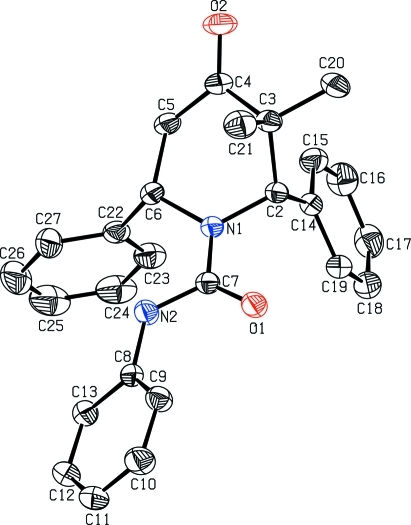
The ORTEP plot of the molecule with 30% probability displacement ellipsoids.

**Fig. 2.**
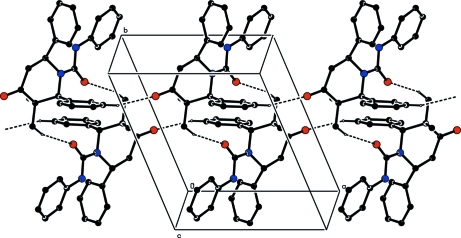
The crystal packing of the molecules viewed along a - axis.

## supplementary crystallographic information

### Comment

Several 2,6-disubstituted piperidine derivatives have fungicidal, herbicidal and bactericidal properties. Both the natural and synthetic piperidine derivatives exhibit high pharmaceutical values (Mobio *et al.*, 1989). Piperidines have a favourable pharmacokinetic profile in rodents and primates, excellent oral bioavailability, and potent antiviral activity against a wide range of primary HIV-1 isolates and considered as promising new candidate for the treatment of HIV-1 infection (Palani  *et al.*, 2002).

The ORTEP plot of the molecule is shown in Fig. 1. The piperidine ring adopts distorted boat conformation, with the puckering amplitudes q2 = 0.5877 (13)°, q3 =-0.1100 (13)°, φ = 258.06 (12)° (Cremer & Pople, 1975) and asymmetry parameters Δ_s_(C2)=Δ_s_(C5) = 18.55 (12)° (Nardelli, 1983). The planar phenyl rings substituted at positions 2 and 6 occupy axial [70.71 (13)°] and equatorial [-162.73 (11)°] orientations and are approximately perpendicular to each other [89.14 (8)°]. One of the methyl groups attached at position 3 of the piperidine ring occupy equatorial [N1-C2-C3-C20 = 177.95 (10)°] orientation and other one is in axial [N1-C2-C3-C21 = 58.31 (12)°] orientation. The sum of the bond angles around N1[358.2 (3)°] indicates *sp^2^* hybridization (Beddoes  *et al.*, 1986).

The phenylcarbamoyl group attached to the N1 atom adopts an extended conformation which is evidenced from the torsion angle [N1-C7-N2-C8=]169.19 (11)°. The crystal structure is stabilized by C-H···O type of intermolecular interactions in addition to van der Waals forces. The C20-H20B···O1 interaction between the molecules lead to the dimer arrangement along the *bc* plane. These dimers are interconnected by C18-H18···O2 hydrogen bonds, which form a one dimensional chain running along *a* - axis (Fig. 2).

### Experimental

A mixture of c-3,t-3-dimethyl-r-2,c-6-diphenylpiperidin-4-one (1.4g), phenylisocyanate (1.1ml) and triethylamine (2ml) in anhydrous benzene (20ml) was stirred at room temperature for 7 hours. The precipitated ammonium salt was washed with water (40ml). The resulting pasty mass was purified by crystallization from benzene and pet-ether (60-80°C) in the ratio of 95 : 5.

### Refinement

H atoms were positioned geometrically (C-H = 0.93 - 0.98Å) and allowed to ride on their parent atoms, with *U*_iso_(H) = 1.5*U*_eq_(C) for methyl H and 1.2*U*_eq_(C) for other H atoms. The components of the anisotropic displacement parameters of C24 and C25 in the direction of the bond between them were restrained to be equal within an effective standard deviation of 0.001.

### Crystal data

**Table:**

C_26_H_26_N_2_O_2_	*Z* = 2
*M**_r_* = 398.49	*F*(000) = 424
Triclinic, *P*1	*D*_x_ = 1.237 Mg m^−^^3^
Hall symbol: -P 1	Mo *K*α radiation, λ = 0.71073 Å
*a* = 9.6648 (2) Å	Cell parameters from 6362 reflections
*b* = 10.7938 (3) Å	θ = 2.1–30.3°
*c* = 11.4233 (3) Å	µ = 0.08 mm^−^^1^
α = 101.303 (2)°	*T* = 293 K
β = 90.158 (1)°	Block, colourless
γ = 113.191 (1)°	0.12 × 0.12 × 0.10 mm
*V* = 1069.91 (5) Å^3^	

### Data collection

**Table:**

Bruker Kappa APEXII area-detector diffractometer	6362 independent reflections
Radiation source: fine-focus sealed tube	4483 reflections with *I* > 2σ(*I*)
graphite	*R*_int_ = 0.027
ω and φ scans	θ_max_ = 30.3°, θ_min_ = 2.1°
Absorption correction: multi-scan (*SADABS*; Sheldrick, 2001)	*h* = −13→13
*T*_min_ = 0.991, *T*_max_ = 0.992	*k* = −15→15
26776 measured reflections	*l* = −16→16

### Refinement

**Table:**

Refinement on *F*^2^	Secondary atom site location: difference Fourier map
Least-squares matrix: full	Hydrogen site location: inferred from neighbouring sites
*R*[*F*^2^ > 2σ(*F*^2^)] = 0.045	H atoms treated by a mixture of independent and constrained refinement
*wR*(*F*^2^) = 0.129	*w* = 1/[σ^2^(*F*_o_^2^) + (0.0562*P*)^2^ + 0.1478*P*] where *P* = (*F*_o_^2^ + 2*F*_c_^2^)/3
*S* = 1.03	(Δ/σ)_max_ = 0.010
6362 reflections	Δρ_max_ = 0.23 e Å^−^^3^
278 parameters	Δρ_min_ = −0.16 e Å^−^^3^
1 restraint	Extinction correction: *SHELXS97* (Sheldrick, 2008), Fc^*^=kFc[1+0.001xFc^2^λ^3^/sin(2θ)]^-1/4^
Primary atom site location: structure-invariant direct methods	Extinction coefficient: 0.034 (3)

### Special details

**Table:**

Geometry. All esds (except the esd in the dihedral angle between two l.s. planes) are estimated using the full covariance matrix. The cell esds are taken into account individually in the estimation of esds in distances, angles and torsion angles; correlations between esds in cell parameters are only used when they are defined by crystal symmetry. An approximate (isotropic) treatment of cell esds is used for estimating esds involving l.s. planes.
Refinement. Refinement of F^2^ against ALL reflections. The weighted R-factor wR and goodness of fit S are based on F^2^, conventional R-factors R are based on F, with F set to zero for negative F^2^. The threshold expression of F^2^ > 2sigma(F^2^) is used only for calculating R-factors(gt) etc. and is not relevant to the choice of reflections for refinement. R-factors based on F^2^ are statistically about twice as large as those based on F, and R- factors based on ALL data will be even larger.

### Fractional atomic coordinates and isotropic or equivalent isotropic displacement parameters (Å^2^)

**Table:**

	*x*	*y*	*z*	*U*_iso_*/*U*_eq_	
C2	0.44729 (12)	0.65312 (11)	0.17958 (10)	0.0397 (2)	
H2	0.4661	0.5889	0.1148	0.048*	
C3	0.27465 (12)	0.60768 (12)	0.17189 (11)	0.0464 (3)	
C4	0.23264 (13)	0.71273 (13)	0.25470 (11)	0.0482 (3)	
C5	0.34974 (13)	0.85807 (13)	0.28686 (12)	0.0486 (3)	
H5A	0.3981	0.8718	0.3656	0.058*	
H5B	0.2979	0.9197	0.2936	0.058*	
C6	0.47423 (12)	0.90306 (11)	0.20204 (10)	0.0414 (2)	
H6	0.4324	0.9268	0.1356	0.050*	
C7	0.60634 (12)	0.79834 (11)	0.05299 (10)	0.0397 (2)	
C8	0.78032 (12)	0.96713 (11)	−0.05439 (10)	0.0404 (2)	
C9	0.77964 (15)	0.88317 (13)	−0.16236 (11)	0.0503 (3)	
H9	0.7057	0.7938	−0.1843	0.060*	
C10	0.89077 (17)	0.93379 (15)	−0.23770 (13)	0.0628 (4)	
H10	0.8916	0.8770	−0.3101	0.075*	
C11	0.99929 (16)	1.06556 (15)	−0.20790 (14)	0.0628 (4)	
H11	1.0729	1.0980	−0.2596	0.075*	
C12	0.99842 (15)	1.14898 (14)	−0.10158 (14)	0.0582 (3)	
H12	1.0711	1.2390	−0.0812	0.070*	
C13	0.89053 (14)	1.10043 (12)	−0.02441 (11)	0.0485 (3)	
H13	0.8916	1.1575	0.0484	0.058*	
C14	0.52831 (12)	0.64977 (11)	0.29232 (10)	0.0419 (2)	
C15	0.48157 (15)	0.66344 (14)	0.40654 (12)	0.0550 (3)	
H15	0.3888	0.6690	0.4183	0.066*	
C16	0.5715 (2)	0.66892 (17)	0.50407 (14)	0.0684 (4)	
H16	0.5387	0.6785	0.5805	0.082*	
C17	0.70797 (18)	0.66035 (16)	0.48857 (15)	0.0683 (4)	
H17	0.7680	0.6646	0.5543	0.082*	
C18	0.75602 (15)	0.64543 (15)	0.37601 (15)	0.0630 (4)	
H18	0.8486	0.6392	0.3651	0.076*	
C19	0.66678 (13)	0.63960 (13)	0.27891 (13)	0.0510 (3)	
H19	0.6999	0.6286	0.2027	0.061*	
C20	0.19496 (16)	0.46380 (14)	0.19745 (15)	0.0632 (4)	
H20A	0.2215	0.4659	0.2791	0.095*	
H20B	0.2257	0.4003	0.1447	0.095*	
H20C	0.0876	0.4347	0.1847	0.095*	
C21	0.22210 (16)	0.60584 (16)	0.04407 (13)	0.0628 (4)	
H21A	0.1147	0.5793	0.0374	0.094*	
H21B	0.2470	0.5409	−0.0124	0.094*	
H21C	0.2718	0.6962	0.0275	0.094*	
C22	0.60341 (13)	1.03354 (12)	0.26859 (11)	0.0474 (3)	
C23	0.70016 (15)	1.02965 (15)	0.35611 (12)	0.0587 (3)	
H23	0.6878	0.9455	0.3737	0.070*	
C24	0.81482 (18)	1.1497 (2)	0.41752 (16)	0.0796 (5)	
H24	0.8786	1.1460	0.4766	0.095*	
C25	0.8349 (2)	1.27372 (19)	0.3918 (2)	0.0935 (6)	
H25	0.9122	1.3543	0.4334	0.112*	
C26	0.7412 (2)	1.27938 (16)	0.3048 (2)	0.0903 (6)	
H26	0.7558	1.3639	0.2870	0.108*	
C27	0.62430 (18)	1.15950 (14)	0.24296 (15)	0.0664 (4)	
H27	0.5603	1.1640	0.1845	0.080*	
N1	0.52148 (10)	0.79071 (9)	0.15014 (8)	0.0386 (2)	
N2	0.67000 (12)	0.92692 (11)	0.02715 (9)	0.0469 (2)	
O1	0.62406 (11)	0.69939 (9)	−0.00520 (8)	0.0565 (2)	
O2	0.11166 (10)	0.68212 (11)	0.29595 (10)	0.0686 (3)	
H2A	0.6752 (17)	0.9942 (16)	0.0847 (14)	0.061 (4)*	

### Atomic displacement parameters (Å^2^)

**Table:**

	*U*^11^	*U*^22^	*U*^33^	*U*^12^	*U*^13^	*U*^23^
C2	0.0345 (5)	0.0362 (5)	0.0476 (6)	0.0130 (4)	0.0116 (4)	0.0097 (4)
C3	0.0332 (5)	0.0434 (6)	0.0576 (7)	0.0109 (4)	0.0071 (5)	0.0091 (5)
C4	0.0336 (5)	0.0558 (7)	0.0562 (7)	0.0185 (5)	0.0092 (5)	0.0132 (5)
C5	0.0407 (6)	0.0486 (6)	0.0588 (7)	0.0219 (5)	0.0163 (5)	0.0078 (5)
C6	0.0381 (5)	0.0406 (5)	0.0475 (6)	0.0186 (4)	0.0088 (4)	0.0081 (4)
C7	0.0360 (5)	0.0403 (5)	0.0425 (5)	0.0149 (4)	0.0079 (4)	0.0090 (4)
C8	0.0395 (5)	0.0421 (6)	0.0443 (6)	0.0190 (5)	0.0088 (4)	0.0145 (4)
C9	0.0519 (7)	0.0447 (6)	0.0498 (6)	0.0154 (5)	0.0129 (5)	0.0084 (5)
C10	0.0709 (9)	0.0624 (8)	0.0555 (8)	0.0271 (7)	0.0264 (7)	0.0123 (6)
C11	0.0537 (8)	0.0642 (9)	0.0750 (9)	0.0229 (7)	0.0297 (7)	0.0265 (7)
C12	0.0436 (7)	0.0491 (7)	0.0772 (9)	0.0112 (5)	0.0121 (6)	0.0187 (6)
C13	0.0466 (6)	0.0443 (6)	0.0531 (7)	0.0171 (5)	0.0063 (5)	0.0099 (5)
C14	0.0374 (5)	0.0363 (5)	0.0525 (6)	0.0135 (4)	0.0101 (5)	0.0134 (4)
C15	0.0532 (7)	0.0634 (8)	0.0559 (7)	0.0281 (6)	0.0154 (6)	0.0192 (6)
C16	0.0810 (10)	0.0730 (10)	0.0530 (8)	0.0312 (8)	0.0074 (7)	0.0168 (7)
C17	0.0655 (9)	0.0618 (9)	0.0740 (10)	0.0196 (7)	−0.0122 (7)	0.0197 (7)
C18	0.0425 (7)	0.0569 (8)	0.0917 (11)	0.0185 (6)	0.0011 (7)	0.0237 (7)
C19	0.0410 (6)	0.0506 (7)	0.0660 (8)	0.0195 (5)	0.0136 (5)	0.0199 (6)
C20	0.0460 (7)	0.0478 (7)	0.0863 (10)	0.0084 (6)	0.0184 (7)	0.0154 (7)
C21	0.0466 (7)	0.0664 (9)	0.0634 (8)	0.0150 (6)	−0.0062 (6)	0.0037 (7)
C22	0.0423 (6)	0.0429 (6)	0.0523 (6)	0.0161 (5)	0.0160 (5)	0.0012 (5)
C23	0.0482 (7)	0.0603 (8)	0.0567 (7)	0.0163 (6)	0.0078 (6)	0.0002 (6)
C24	0.0541 (8)	0.0866 (10)	0.0687 (10)	0.0134 (8)	0.0045 (7)	−0.0170 (8)
C25	0.0692 (11)	0.0643 (9)	0.1014 (14)	0.0008 (8)	0.0184 (10)	−0.0282 (8)
C26	0.0941 (13)	0.0401 (8)	0.1177 (16)	0.0155 (8)	0.0353 (12)	−0.0011 (8)
C27	0.0710 (9)	0.0430 (7)	0.0821 (10)	0.0227 (6)	0.0206 (8)	0.0062 (6)
N1	0.0362 (4)	0.0361 (4)	0.0446 (5)	0.0151 (4)	0.0113 (4)	0.0094 (4)
N2	0.0549 (6)	0.0398 (5)	0.0472 (5)	0.0194 (4)	0.0190 (4)	0.0108 (4)
O1	0.0640 (6)	0.0426 (5)	0.0640 (5)	0.0224 (4)	0.0311 (4)	0.0117 (4)
O2	0.0388 (5)	0.0738 (7)	0.0890 (7)	0.0197 (4)	0.0237 (5)	0.0137 (5)

### Geometric parameters (Å, °)

**Table:**

C2—N1	1.4802 (13)	C14—C15	1.3816 (17)
C2—C14	1.5193 (16)	C14—C19	1.3917 (16)
C2—C3	1.5395 (15)	C15—C16	1.389 (2)
C2—H2	0.9800	C15—H15	0.9300
C3—C4	1.5132 (17)	C16—C17	1.368 (2)
C3—C20	1.5256 (18)	C16—H16	0.9300
C3—C21	1.5384 (19)	C17—C18	1.370 (2)
C4—O2	1.2075 (14)	C17—H17	0.9300
C4—C5	1.5001 (17)	C18—C19	1.379 (2)
C5—C6	1.5339 (15)	C18—H18	0.9300
C5—H5A	0.9700	C19—H19	0.9300
C5—H5B	0.9700	C20—H20A	0.9600
C6—N1	1.4786 (13)	C20—H20B	0.9600
C6—C22	1.5201 (16)	C20—H20C	0.9600
C6—H6	0.9800	C21—H21A	0.9600
C7—O1	1.2159 (13)	C21—H21B	0.9600
C7—N2	1.3715 (15)	C21—H21C	0.9600
C7—N1	1.3779 (13)	C22—C27	1.3839 (19)
C8—C9	1.3783 (16)	C22—C23	1.385 (2)
C8—C13	1.3856 (16)	C23—C24	1.381 (2)
C8—N2	1.4115 (14)	C23—H23	0.9300
C9—C10	1.3856 (17)	C24—C25	1.366 (3)
C9—H9	0.9300	C24—H24	0.9300
C10—C11	1.368 (2)	C25—C26	1.369 (3)
C10—H10	0.9300	C25—H25	0.9300
C11—C12	1.365 (2)	C26—C27	1.392 (2)
C11—H11	0.9300	C26—H26	0.9300
C12—C13	1.3765 (18)	C27—H27	0.9300
C12—H12	0.9300	N2—H2A	0.864 (16)
C13—H13	0.9300		
			
N1—C2—C14	109.54 (9)	C14—C15—C16	120.74 (13)
N1—C2—C3	110.26 (9)	C14—C15—H15	119.6
C14—C2—C3	119.32 (9)	C16—C15—H15	119.6
N1—C2—H2	105.6	C17—C16—C15	120.48 (14)
C14—C2—H2	105.6	C17—C16—H16	119.8
C3—C2—H2	105.6	C15—C16—H16	119.8
C4—C3—C20	111.63 (10)	C16—C17—C18	119.82 (14)
C4—C3—C21	106.12 (11)	C16—C17—H17	120.1
C20—C3—C21	109.08 (11)	C18—C17—H17	120.1
C4—C3—C2	110.60 (9)	C17—C18—C19	119.84 (13)
C20—C3—C2	111.34 (10)	C17—C18—H18	120.1
C21—C3—C2	107.87 (10)	C19—C18—H18	120.1
O2—C4—C5	120.53 (11)	C18—C19—C14	121.52 (13)
O2—C4—C3	122.26 (11)	C18—C19—H19	119.2
C5—C4—C3	117.19 (9)	C14—C19—H19	119.2
C4—C5—C6	117.78 (10)	C3—C20—H20A	109.5
C4—C5—H5A	107.9	C3—C20—H20B	109.5
C6—C5—H5A	107.9	H20A—C20—H20B	109.5
C4—C5—H5B	107.9	C3—C20—H20C	109.5
C6—C5—H5B	107.9	H20A—C20—H20C	109.5
H5A—C5—H5B	107.2	H20B—C20—H20C	109.5
N1—C6—C22	113.61 (9)	C3—C21—H21A	109.5
N1—C6—C5	112.05 (9)	C3—C21—H21B	109.5
C22—C6—C5	108.49 (9)	H21A—C21—H21B	109.5
N1—C6—H6	107.5	C3—C21—H21C	109.5
C22—C6—H6	107.5	H21A—C21—H21C	109.5
C5—C6—H6	107.5	H21B—C21—H21C	109.5
O1—C7—N2	122.47 (10)	C27—C22—C23	118.93 (13)
O1—C7—N1	122.90 (10)	C27—C22—C6	119.72 (13)
N2—C7—N1	114.63 (9)	C23—C22—C6	121.35 (12)
C9—C8—C13	119.39 (10)	C24—C23—C22	120.53 (16)
C9—C8—N2	123.48 (10)	C24—C23—H23	119.7
C13—C8—N2	117.08 (10)	C22—C23—H23	119.7
C8—C9—C10	119.03 (12)	C25—C24—C23	120.28 (19)
C8—C9—H9	120.5	C25—C24—H24	119.9
C10—C9—H9	120.5	C23—C24—H24	119.9
C11—C10—C9	121.42 (13)	C24—C25—C26	119.99 (16)
C11—C10—H10	119.3	C24—C25—H25	120.0
C9—C10—H10	119.3	C26—C25—H25	120.0
C12—C11—C10	119.36 (12)	C25—C26—C27	120.40 (18)
C12—C11—H11	120.3	C25—C26—H26	119.8
C10—C11—H11	120.3	C27—C26—H26	119.8
C11—C12—C13	120.31 (12)	C22—C27—C26	119.87 (18)
C11—C12—H12	119.8	C22—C27—H27	120.1
C13—C12—H12	119.8	C26—C27—H27	120.1
C12—C13—C8	120.47 (12)	C7—N1—C6	121.29 (9)
C12—C13—H13	119.8	C7—N1—C2	116.56 (8)
C8—C13—H13	119.8	C6—N1—C2	120.31 (8)
C15—C14—C19	117.59 (12)	C7—N2—C8	125.58 (9)
C15—C14—C2	126.22 (10)	C7—N2—H2A	115.1 (10)
C19—C14—C2	116.09 (10)	C8—N2—H2A	113.3 (10)
			
N1—C2—C3—C4	−57.33 (13)	C16—C17—C18—C19	0.2 (2)
C14—C2—C3—C4	70.71 (13)	C17—C18—C19—C14	0.6 (2)
N1—C2—C3—C20	177.95 (10)	C15—C14—C19—C18	−1.17 (18)
C14—C2—C3—C20	−54.01 (14)	C2—C14—C19—C18	175.36 (11)
N1—C2—C3—C21	58.31 (12)	N1—C6—C22—C27	129.49 (12)
C14—C2—C3—C21	−173.65 (10)	C5—C6—C22—C27	−105.18 (13)
C20—C3—C4—O2	−29.31 (18)	N1—C6—C22—C23	−51.43 (14)
C21—C3—C4—O2	89.41 (15)	C5—C6—C22—C23	73.89 (13)
C2—C3—C4—O2	−153.86 (13)	C27—C22—C23—C24	0.55 (19)
C20—C3—C4—C5	149.20 (12)	C6—C22—C23—C24	−178.53 (12)
C21—C3—C4—C5	−92.08 (13)	C22—C23—C24—C25	−0.6 (2)
C2—C3—C4—C5	24.65 (15)	C23—C24—C25—C26	0.0 (3)
O2—C4—C5—C6	−158.95 (12)	C24—C25—C26—C27	0.7 (3)
C3—C4—C5—C6	22.51 (17)	C23—C22—C27—C26	0.1 (2)
C4—C5—C6—N1	−36.49 (15)	C6—C22—C27—C26	179.15 (13)
C4—C5—C6—C22	−162.73 (11)	C25—C26—C27—C22	−0.7 (2)
C13—C8—C9—C10	0.85 (19)	O1—C7—N1—C6	−166.29 (11)
N2—C8—C9—C10	178.19 (12)	N2—C7—N1—C6	13.92 (15)
C8—C9—C10—C11	−1.0 (2)	O1—C7—N1—C2	−1.72 (16)
C9—C10—C11—C12	0.1 (2)	N2—C7—N1—C2	178.49 (10)
C10—C11—C12—C13	0.8 (2)	C22—C6—N1—C7	−71.04 (14)
C11—C12—C13—C8	−0.9 (2)	C5—C6—N1—C7	165.56 (10)
C9—C8—C13—C12	0.09 (19)	C22—C6—N1—C2	124.96 (11)
N2—C8—C13—C12	−177.42 (12)	C5—C6—N1—C2	1.56 (14)
N1—C2—C14—C15	100.75 (13)	C14—C2—N1—C7	107.12 (10)
C3—C2—C14—C15	−27.62 (17)	C3—C2—N1—C7	−119.65 (10)
N1—C2—C14—C19	−75.44 (12)	C14—C2—N1—C6	−88.14 (11)
C3—C2—C14—C19	156.19 (10)	C3—C2—N1—C6	45.08 (13)
C19—C14—C15—C16	1.00 (19)	O1—C7—N2—C8	−10.60 (19)
C2—C14—C15—C16	−175.14 (12)	N1—C7—N2—C8	169.19 (11)
C14—C15—C16—C17	−0.2 (2)	C9—C8—N2—C7	39.03 (18)
C15—C16—C17—C18	−0.4 (2)	C13—C8—N2—C7	−143.58 (12)

### Hydrogen-bond geometry (Å, °)

**Table:**

*D*—H···*A*	*D*—H	H···*A*	*D*···*A*	*D*—H···*A*
C18—H18···O2^i^	0.93	2.56	3.4488 (17)	161
C20—H20B···O1^ii^	0.96	2.52	3.4520 (17)	162

Symmetry codes: (i) *x*+1, *y*, *z*; (ii) −*x*+1, −*y*+1, −*z*.
